# Use of three-dimensional time-resolved phase-contrast magnetic resonance imaging with vastly undersampled isotropic projection reconstruction to assess renal blood flow in a renal cell carcinoma patient treated with sunitinib: a case report

**DOI:** 10.1186/1756-0500-7-527

**Published:** 2014-08-14

**Authors:** Tatsuya Takayama, Yasuo Takehara, Masataka Sugiyama, Takayuki Sugiyama, Yasuo Ishii, Kevin E Johnson, Oliver Wieben, Tetsuya Wakayama, Harumi Sakahara, Seiichiro Ozono

**Affiliations:** Departments of Urology, Hamamatsu University School of Medicine, 1-20-1 Higashi-ku, Handayama, Hamamatsu, Shizuoka, 431-3192 Japan; Departments of Radiology, Hamamatsu University School of Medicine, 1-20-1 Higashi-ku, Handayama, Hamamatsu, Shizuoka, 431-3192 Japan; Department of Medical Physics and Radiology, University of Wisconsin-Madison, 600 Highland Ave, CSC, Madison, WI 53792 USA; Applied Science Laboratory Asia Pacific, GE Healthcare Japan, 4-7-127, Asahigaoka, Hino, Tokyo, 191-8503 Japan

**Keywords:** Renal cell carcinoma, Magnetic resonance, Phase contrast imaging, Flow analysis, Sunitinib

## Abstract

**Background:**

New imaging modalities to assess the efficacy of drugs that have molecular targets remain under development. Here, we describe for the first time the use of time-resolved three-dimensional phase-contrast magnetic resonance imaging to monitor changes in blood supply to a tumor during sunitinib treatment in a patient with localized renal cell carcinoma.

**Case presentation:**

A 43-year-old Japanese woman with a tumor-bearing but functional single kidney presented at our hospital in July 2012. Computed tomography and magnetic resonance imaging revealed a cT1aN0M0 renal cell carcinoma embedded in the upper central region of the left kidney. She was prescribed sunitinib as neoadjuvant therapy for 8 months, and then underwent partial nephrectomy. Tumor monitoring during this time was done using time-resolved three-dimensional phase-contrast magnetic resonance imaging, a recent technique which specifically measures blood flow in the various vessels of the kidney. This imaging allowed visualization of the redistribution of renal blood flow during treatment, and showed that flow to the tumor was decreased and flows to other areas increased. Of note, this change occurred in the absence of any change in tumor size.

**Conclusion:**

The ability of time-resolved three-dimensional phase-contrast magnetic resonance imaging to provide quantitative information on blood supply to tumors may be useful in monitoring the efficacy of sunitinib treatment.

## Background

The introduction of drugs targeting specific molecules has markedly changed the treatment of advanced renal cell carcinoma (RCC) [1–4]. Drugs currently approved in Japan include tyrosine kinase inhibitors (TKIs) such as sorafenib, sunitinib and axitinib, and mammalian target of rapamycin (mTOR) inhibitors, including everolimus and temsirolimus. With ongoing improvement in the resolution of imaging modalities, including multidetector row computed tomography (CT) and high-tesla magnetic resonance imaging (MRI), more small renal masses are diagnosed incidentally, and surgical management has shifted from radical toward partial nephrectomy. Moreover, the extended use of TKIs and mTOR inhibitors to reduce primary tumor volume, followed by application of neoadjuvant therapy, is now widely used as an organ-sparing strategy for RCC patients. In addition, drugs that have molecular targets are increasingly assessed using the Choi criteria [5], and novel criteria that can be applied after evaluation with fluorodeoxyglucose-positron emission tomography [6, 7]. To date, however, few imaging modalities have been used in this context. To our knowledge, this paper is the first to show that 3D PC VIPR can be used to monitor changes in blood supply to a tumor during drug treatment.

## Case presentation

**Figure 1:**
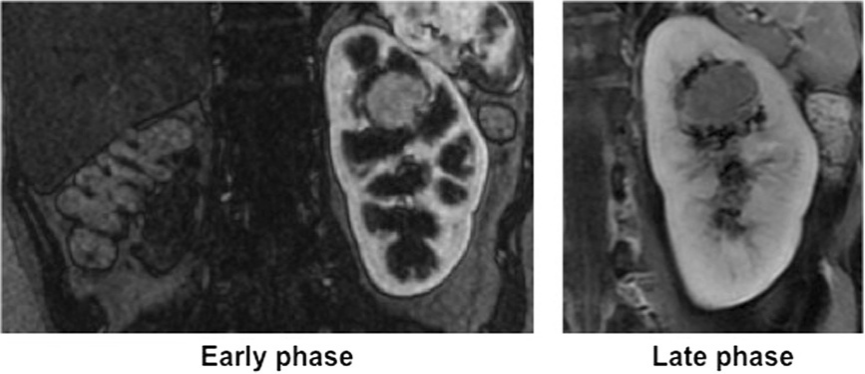
**Contrast-enhanced coronal T1-weighted imaging with fat saturation (left: arterial phase; right: late phase) reveals a hypervascular solitary solid mass in the upper half of the left (functional) kidney.**

A 43-year-old Japanese woman with a tumor-bearing but functional single left kidney presented at our hospital in July 2012. Her past history and family history were unremarkable in the present context. CT and MRI revealed a cT1aN0M0 RCC with maximum scores in preoperative aspects, anatomical Preoperative Aspects and Dimensions Used for an Anatomical (PADUA) criteria, and the radius exophytic/endophytic nearness anterior/posterior location (RENAL) criteria used to grade renal neoplasms (Figure 1). The tumor was embedded in the upper central region of the kidney. Because she had only a single functioning kidney, only partial nephrectomy was appropriate. However, the tumor location suggested the possibility of severe complications even after partial nephrectomy. We therefore commenced treatment with sunitinib as neoadjuvant therapy, and used MRI to monitor her condition.

**Figure 2:**
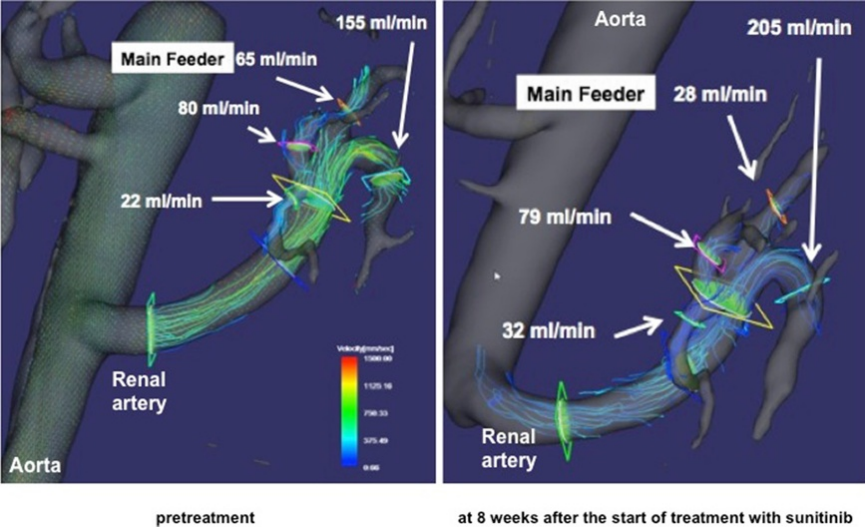
**Blood flow in arterial branches visualized using time-resolved three-dimensional phase-contrast magnetic resonance imaging.** Flows are visualized as streamlines. The regions of branches where flows were measured are shown. Blood flow in the main feeder to the tumor was 65 ml/min (left) before sunitinib treatment, but decreased to 28 ml/min after 8 weeks of sunitinib treatment (right). Of note, flow rates in the other branches increased simultaneously, which may indicate flow redistribution from the tumor to other kidney segments.

The MRI modalities employed ECG-gated, respiration-compensated, time-resolved respiratory-gated three-dimensional (3D) phase-contrast (PC) MRI featuring PC-VIPR (phase-contrast vastly undersampled isotropic projection reconstruction) [8]. 3D PC VIPR was performed using a 3.0 Tesla (T) MR scanner (750 Discovery, GEHC, WI) fitted with a 16-channel torso phased-array coil (GEHC), covering the entire left renal artery and parent artery (i.e. abdominal aorta) at the same level. Fast GRE-based sequence was used with the following parameters: field of view (FOV) 32 cm, slice thickness 10 mm, locs/slab 8, repetition time (TR) 7.0–7.1 ms, echo time (TE) 3.3–3.4 ms, flip angle (FA; degrees) 8°, matrix 256 × 256, receiver bandwidth 62.5 kHz, partition thickness 1.25 mm, NEX 1, number of phases/location 20, imaging time 9:29–9:36 min, respiratory window 50%, number of projections 10,000, and encoding velocity (VENC) 100–200 cm/s depending on the maximum velocities measured in preceding 2D cine PC studies. The 4D datasets were post-processed using flow analysis software (flova; R’s Tech Co., Hamamatsu, Japan). A valuable feature of 3D PC VIPR is its capacity to delineate renal branches and obtain flow velocity data without administration of contrast medium (Figure 2).

**Figure 3:**
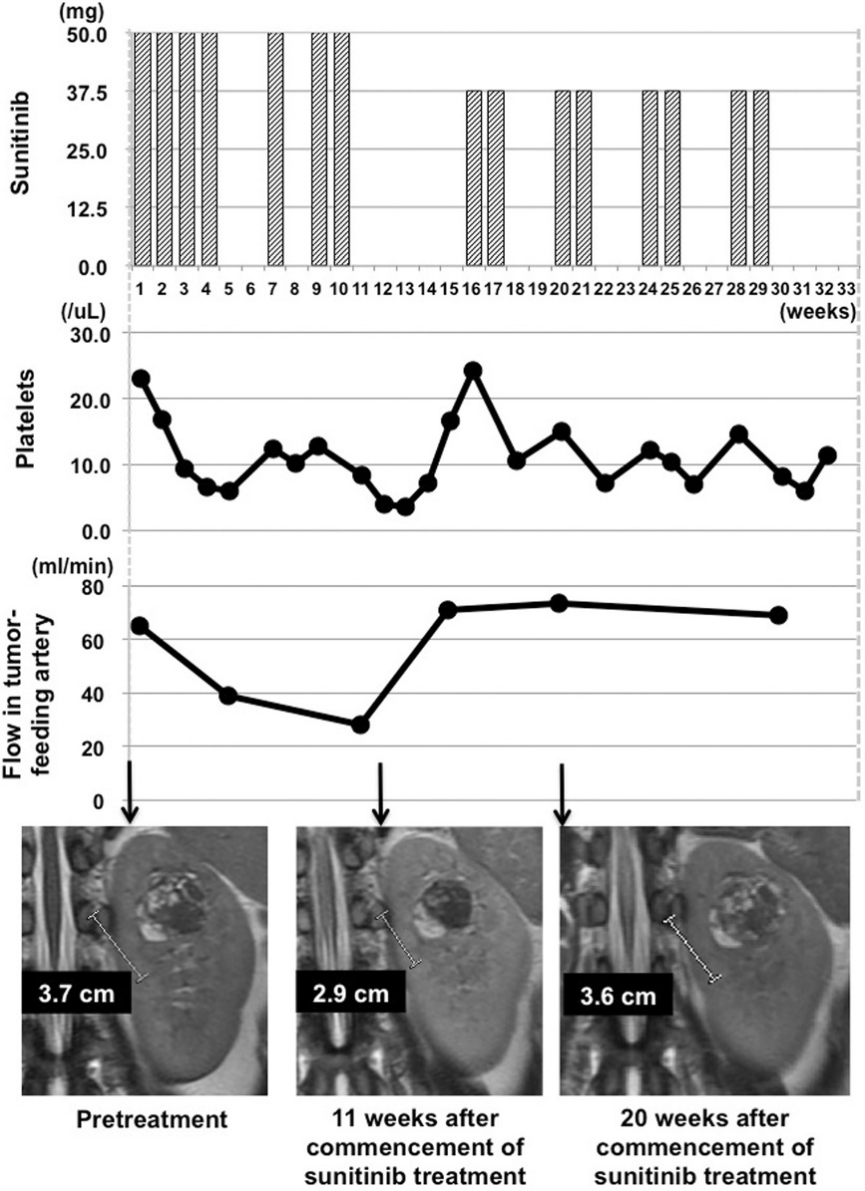
**Clinical course during sunitinib treatment prior to partial nephrectomy.**

Figure 3 illustrates the clinical course of the patient. Sunitinib was administered at the recommended dose (50 mg daily; 4 weeks on followed by 2 weeks off) in the first course and at 75% relative dose intensity in the second. Tumor size and density measured on CT decreased slightly, and disease status was stable according to Response Evaluation Criteria in Solid Tumors version 1.0 (RECIST v1.0). Tumor angiogenicity was disrupted by sunitinib, and renal blood flow was redistributed with a decrease in blood flow to the tumor and increase to other areas. Platelet concentration gradually decreased and she was classified as grade 3 under the Common Terminology Criteria Adverse Events version 3.0 system. She also developed grade 2 hypertension, grade 2 hand-foot skin reactions, grade 1 dysphonia, grade 1 proteinuria, and grade 2 hypothyroidism. Sunitinib treatment was thus modified to four cycles of 2 weeks on sunitinib (37.5 mg daily) followed by a 2-week drug holiday in an effort to restore platelet levels and achieve resolution of other adverse events. However, blood flow to the tumor and tumor size reverted to and remained at levels before sunitinib treatment (Figure 3). We judged that sunitinib was unlikely to yield further useful effects, and treatment was discontinued. The patient then underwent partial nephrectomy 1 month later. We could not find any tumor necrosis or hemorrhage reflecting the effect of sunitinib treatment pathologically. Both the operative and postoperative courses were uneventful, and she remains in good condition, with no tumor recurrence, an estimated glomerular filtration rate of 47 ml/min/1.73 m^2^, and grade 2 hypothyroidism.

## Conclusion

3D PC VIPR is a recently developed phase-contrast MR technique which uses a new k-space trajectory. A major advantage of 3D PC VIPR is its ability to both morphologically image the renal arteries and veins, and measure their blood flow velocity. Use of flow analysis software then permits flow streamlines to be visualized. A second major advantage of 3D PC VIPR is that it does not require contrast medium for renal artery depiction. Many patients with renal neoplasia have impaired renal function, and clinicians are now reluctant to administer gadolinium chelate contrast media to these patients due to the risk of nephrogenic systemic fibrosis [9].

Here, we describe a patient with RCC who underwent partial nephrectomy 8 months after the commencement of sunitinib as neoadjuvant therapy with monitoring using 3D PC VIPR. Of note, total renal arterial flow level barely changed over the entire course of treatment. Sunitinib (one course of the full recommended dose and a second course at 75% of that dose) reduced blood flow to the tumor and increased flow to other areas of the kidney. When sunitinib was recommenced after cessation to allow recovery from adverse effects, however, it did not induce the same redistribution of blood flow as it had before cessation. It is notable that the redistribution of renal blood flow might be associated with the induction of stable disease, as identified using the RECIST criteria. Although it is unclear whether this was caused by the reduction in sunitinib dose or acquisition of drug resistance by the tumor, we emphasize that 3D PC VIPR was markedly useful in monitoring therapeutic response as it allowed the measurement of blood flow in the tumor feeder vessel. These findings suggest that we should have conducted partial nephrectomy without additional administration of sunitinib at any time from 20 weeks onward.

One limitation of 3D PC-VIPR is that it is difficult to measure blood flow in very small arteries, such as tertiary vessels or smaller, using prescribed velocity encoding.

Non-contrast 3D PC VIPR revealed the redistribution of renal blood flow in a patient with RCC treated with sunitinib, including that to the tumor, without any change in tumor size. This modality may be useful in predicting the efficacy of sunitinib in terms of blood flow redistribution, and in determining surgical timing. Further evaluation in a large number of cases is warranted.

## Consent

Written informed consent was obtained from the patient for publication of this case report and any accompanying images. A copy of the written consent is available for review by the Editor of this journal.

## Abbreviations

RCC: Renal cell carcinoma
TKIs: Tyrosine kinase inhibitors
mTOR: Mammalian target of rapamycin
CT: Computed tomography
MRI: Magnetic resonance imaging
3D: Three-dimensional
PC: Phase-contrast
PC-VIPR: Phase-contrast vastly undersampled isotropic projection reconstruction
RECIST v1.0: Response Evaluation Criteria in Solid Tumors version 1.0.
